# Supplementary material for: Testing hypothetical bias in a choice experiment: An application to the value of the carbon footprint of mandarin oranges
Source: PLoS One. 2022 Jan 18;17(1):e0261369. doi: 10.1371/journal.pone.0261369 (PMC8765649; doi:10.1371/journal.pone.0261369)
Supplement: S1 Table — (DOCX) [file pone.0261369.s001.docx]

Table. The random parameter logit regression results in the main effect (Model 1) and that with interaction (Model 2) (All)

|  | Non-hypothetical Lab Economic Experiment (NHLEE) | | | | Hypothetical Lab Survey (HLS) | | | | Hypothetical Online Survey (HOS) | | | | Hypothetical Online Survey with Cheap-talk (HOSCT) | | | |
| --- | --- | --- | --- | --- | --- | --- | --- | --- | --- | --- | --- | --- | --- | --- | --- | --- |
|  | Model 1 | | Model 2 | | Model 1 | | Model 2 | | Model 1 | | Model 2 | | Model 1 | | Model 2 | |
| *Random parameter* |  |  |  |  |  |  |  |  |  |  |  |  |  |  |  |  |
| Carbon | -0.08 | *** | -0.09 |  | -0.08 | *** | -0.02 |  | -0.10 | *** | 0.02 |  | -0.10 | *** | -0.06 |  |
|  | (0.01) |  | (0.09) |  | (0.01) |  | (0.05) |  | (0.01) |  | (0.04) |  | (0.01) |  | (0.04) |  |
| Price | -0.15 | *** | -0.20 | * | -0.16 | *** | -0.21 | *** | -0.18 | *** | -0.18 | *** | -0.18 | *** | -0.25 | *** |
|  | (0.02) |  | (0.10) |  | (0.01) |  | (0.07) |  | (0.01) |  | (0.07) |  | (0.01) |  | (0.07) |  |
| *Heterogeneity in mean* | |  |  |  |  |  |  |  |  |  |  |  |  |  |  |  |
| Carbon × Female |  |  | -0.05 |  |  |  | -0.02 |  |  |  | -0.01 |  |  |  | -0.03 | ** |
|  |  |  | (0.03) |  |  |  | (0.02) |  |  |  | (0.01) |  |  |  | (0.01) |  |
| Carbon × Age |  |  | 0.02 | * |  |  | 0.01 | *** |  |  | 0.00 |  |  |  | 0.00 |  |
|  |  |  | (0.01) |  |  |  | (0.00) |  |  |  | (0.00) |  |  |  | (0.00) |  |
| Carbon × Household |  |  | -0.01 |  |  |  | 0.00 |  |  |  | 0.00 |  |  |  | 0.00 |  |
|  |  |  | (0.01) |  |  |  | (0.01) |  |  |  | (0.00) |  |  |  | (0.00) |  |
| Carbon × University |  |  | 0.01 |  |  |  | 0.01 |  |  |  | -0.01 |  |  |  | -0.01 |  |
|  |  |  | (0.03) |  |  |  | (0.01) |  |  |  | (0.01) |  |  |  | (0.01) |  |
| Carbon × Income |  |  | 0.00 |  |  |  | 0.00 |  |  |  | 0.00 |  |  |  | 0.00 |  |
|  |  |  | (0.01) |  |  |  | (0.01) |  |  |  | (0.00) |  |  |  | (0.00) |  |
| Carbon × High ECCB |  |  | -0.04 | * |  |  | -0.03 | ** |  |  | -0.03 | *** |  |  | -0.04 | *** |
|  |  |  | (0.02) |  |  |  | (0.02) |  |  |  | (0.01) |  |  |  | (0.01) |  |
| Carbon × Frequency |  |  | 0.01 |  |  |  | -0.01 |  |  |  | 0.00 |  |  |  | 0.02 | ** |
|  |  |  | (0.02) |  |  |  | (0.01) |  |  |  | (0.01) |  |  |  | (0.01) |  |
| Carbon × Label |  |  | 0.01 |  |  |  | 0.00 |  |  |  | -0.04 | * |  |  | -0.01 |  |
|  |  |  | (0.07) |  |  |  | (0.04) |  |  |  | (0.02) |  |  |  | (0.03) |  |
| Carbon × Not using |  |  | 0.00 |  |  |  | -0.02 |  |  |  | -0.01 |  |  |  | -0.02 | * |
| shopping bags |  |  | (0.02) |  |  |  | (0.02) |  |  |  | (0.01) |  |  |  | (0.01) |  |
| Carbon × Public |  |  | -0.02 |  |  |  | -0.02 |  |  |  | -0.02 |  |  |  | -0.06 | *** |
| Transportation |  |  | (0.02) |  |  |  | (0.02) |  |  |  | (0.01) |  |  |  | (0.02) |  |
| Carbon ×Not tap |  |  | -0.02 |  |  |  | -0.02 |  |  |  | -0.01 |  |  |  | 0.00 |  |
| running |  |  | (0.04) |  |  |  | (0.02) |  |  |  | (0.01) |  |  |  | (0.02) |  |
| Carbon × Air |  |  | 0.00 |  |  |  | 0.01 |  |  |  | -0.01 |  |  |  | -0.02 |  |
| Conditioning |  |  | (0.03) |  |  |  | (0.02) |  |  |  | (0.01) |  |  |  | (0.01) |  |
| Carbon × Walking |  |  | 0.02 |  |  |  | 0.00 |  |  |  | 0.01 |  |  |  | 0.02 |  |
|  |  |  | (0.02) |  |  |  | (0.02) |  |  |  | (0.01) |  |  |  | (0.02) |  |
| Carbon × Garbage |  |  | 0.02 |  |  |  | -0.03 | * |  |  | -0.04 | *** |  |  | -0.01 |  |
|  |  |  | (0.03) |  |  |  | (0.02) |  |  |  | (0.01) |  |  |  | (0.01) |  |
| Price × Female |  |  | 0.06 | * |  |  | 0.03 |  |  |  | 0.05 | ** |  |  | 0.04 | ** |
|  |  |  | (0.03) |  |  |  | (0.03) |  |  |  | (0.02) |  |  |  | (0.02) |  |
| Price × Age |  |  | 0.03 | *** |  |  | 0.01 |  |  |  | 0.02 | *** |  |  | 0.03 | *** |
|  |  |  | (0.01) |  |  |  | (0.01) |  |  |  | (0.01) |  |  |  | (0.01) |  |
| Price × Household |  |  | -0.01 |  |  |  | 0.02 |  |  |  | 0.00 |  |  |  | 0.01 |  |
|  |  |  | (0.01) |  |  |  | (0.01) |  |  |  | (0.01) |  |  |  | (0.01) |  |
| Price × University |  |  | 0.01 |  |  |  | -0.01 |  |  |  | -0.01 |  |  |  | -0.03 | * |
|  |  |  | (0.03) |  |  |  | (0.02) |  |  |  | (0.02) |  |  |  | (0.02) |  |
| Price × Income |  |  | 0.01 |  |  |  | -0.01 |  |  |  | 0.01 | * |  |  | 0.01 |  |
|  |  |  | (0.01) |  |  |  | (0.01) |  |  |  | (0.01) |  |  |  | (0.01) |  |
| Price × High ECCB |  |  | -0.01 |  |  |  | 0.01 |  |  |  | 0.06 | *** |  |  | 0.04 | ** |
|  |  |  | (0.03) |  |  |  | (0.02) |  |  |  | (0.02) |  |  |  | (0.02) |  |
| Price × Frequency |  |  | -0.01 |  |  |  | 0.01 |  |  |  | -0.02 |  |  |  | -0.01 |  |
|  |  |  | (0.02) |  |  |  | (0.02) |  |  |  | (0.01) |  |  |  | (0.01) |  |
| Price × Label |  |  | -0.02 |  |  |  | 0.02 |  |  |  | -0.11 | *** |  |  | -0.01 |  |
|  |  |  | (0.08) |  |  |  | (0.06) |  |  |  | (0.04) |  |  |  | (0.05) |  |
| Price ×Not using |  |  | -0.01 |  |  |  | -0.06 | ** |  |  | -0.03 | * |  |  | -0.07 | *** |
| shopping bags |  |  | (0.03) |  |  |  | (0.02) |  |  |  | (0.02) |  |  |  | (0.02) |  |
| Price ×Public |  |  | -0.04 | * |  |  | 0.02 |  |  |  | -0.02 |  |  |  | -0.06 | ** |
| Transportation |  |  | (0.02) |  |  |  | (0.02) |  |  |  | (0.02) |  |  |  | (0.03) |  |
| Price × Not tap |  |  | 0.00 |  |  |  | 0.03 |  |  |  | -0.07 | *** |  |  | -0.01 |  |
| running |  |  | (0.05) |  |  |  | (0.03) |  |  |  | (0.02) |  |  |  | (0.02) |  |
| Price × Air |  |  | -0.05 |  |  |  | -0.03 |  |  |  | 0.02 |  |  |  | -0.03 |  |
| Conditioning |  |  | (0.03) |  |  |  | (0.03) |  |  |  | (0.02) |  |  |  | (0.02) |  |
| Price × Walking |  |  | 0.00 |  |  |  | 0.02 |  |  |  | 0.00 |  |  |  | 0.04 | * |
|  |  |  | (0.03) |  |  |  | (0.02) |  |  |  | (0.02) |  |  |  | (0.03) |  |
| Price × Garbage |  |  | -0.05 |  |  |  | -0.04 |  |  |  | 0.02 |  |  |  | -0.02 |  |
|  |  |  | (0.04) |  |  |  | (0.03) |  |  |  | (0.02) |  |  |  | (0.02) |  |
| *Standard deviation* |  |  |  |  |  |  |  |  |  |  |  |  |  |  |  |  |
| Carbon | 0.07 | *** | 0.06 | *** | 0.07 | *** | 0.06 | *** | 0.10 | *** | 0.09 | *** | 0.11 | *** | 0.10 | *** |
|  | (0.01) |  | (0.01) |  | (0.01) |  | (0.01) |  | (0.01) |  | (0.01) |  | (0.01) |  | (0.01) |  |
| Price | 0.12 | *** | 0.06 | *** | 0.13 | *** | 0.12 | *** | 0.19 | *** | 0.18 | *** | 0.20 | *** | 0.18 | *** |
|  | (0.02) |  | (0.01) |  | (0.01) |  | (0.01) |  | (0.01) |  | (0.01) |  | (0.01) |  | (0.01) |  |
| Log Likelihood | -1020.29 |  | -852.69 |  | -2110.42 |  |  |  | -4827.05 |  | -4776.35 |  | -4760.48 |  | -4713.43 |  |
| McFadden R2 | 0.25 |  | 0.24 |  | 0.24 |  |  |  | 0.26 |  | 0.27 |  | 0.27 |  | 0.28 |  |
| Observation | 1248 |  |  |  | 2554 |  |  |  | 6000 |  |  |  | 6000 |  |  |  |
| # of respondents | 104 |  |  |  | 212 |  |  |  | 500 |  |  |  | 500 |  |  |  |

Notes: Standard errors are in parentheses. ***, **, and * denote that the parameters are different from zero at the 1%, 5%, 10% significance levels, respectively.
