# Supplementary material for: Testing hypothetical bias in a choice experiment: An application to the value of the carbon footprint of mandarin oranges
Source: PLoS One. 2022 Jan 18;17(1):e0261369. doi: 10.1371/journal.pone.0261369 (PMC8765649; doi:10.1371/journal.pone.0261369)
Supplement: S1 File — (DOCX) [file pone.0261369.s002.docx]

**Instructions used in the NHLEE treatment (original text in Japanese)**

You are participating in an experiment that is designed to study decision making. In this experiment, you will be asked to buy one of three types of Satsuma mandarin oranges, which we will provide. Please read and follow the instructions carefully. In addition, you cannot communicate with others during the experiment or take any remaining Satsuma mandarin oranges with you after the experiment is completed without instructions regarding the same.

**Overview**

This experiment consists of 12 rounds. In each round, you must choose one of three types of Satsuma mandarin oranges, which we will provide, and pay for it with the money given to you.

This particular type of Satsuma mandarin orange is goku-wase. It is cultivated in gardens in Japan and you must have seen it in stores. The experimenters have bought them at Japan Agriculture and some other food stores. At the end of the experiment, the proof of purchase will be shown by the experimenters.

You will receive your earnings in cash, based on the formula below:

Earnings = 12 * {initial income in each round (120 JPY) – the price of the Satsuma mandarin oranges chosen in each round}+ show-up fees (500 JPY)

Moreover, you can take home the 12 pieces of Satsuma mandarin orange which you choose during the experiment.

**Rules**

At the beginning of round 1, you will receive a hypothetical sum of 120 JPY to buy a Satsuma mandarin orange. You will not actually receive that amount in cash in each round. Please imagine that you have 120 JPY in each round when you make your choice.

Next, you will receive a box containing three types of Satsuma mandarin oranges and a record sheet. Verify your seat number and the round number that appear on it.

We will now consider an example of a “record sheet.” This is a record sheet for seat number 1 in round 1. Further, we will explain how to read and fill in the record sheet. The top line, which states “record sheet” and “round 1,” indicates the round number and the first seat, which is located on the left hand side of the room.

The second line indicates three alternatives —Satsuma mandarin orange A, Satsuma mandarin orange B, and Satsuma mandarin orange C.

The third line indicates the price levels of the Satsuma mandarin oranges in JPY per 100 g. The price of Satsuma mandarin oranges in each round of the experiment is less than the money you receive to buy it (i.e., 120 JPY).

The fourth line indicates the CO_2_ emission levels of the three Satsuma mandarin oranges in grams per 100 grams of Satsuma mandarin orange. These figures indicate the CO_2_ emission levels that are produced during the following processes: production, fruit sorting and box packing, transportation and packaging. The CO_2_ emissions contribute to global warming. The amount of the CO_2_ emissions is based on data obtained from the Ministry of Land, Infrastructure, and Transport, The National Institute for Agro-Environmental Science and the Ajinomoto Group in Japan.

The Satsuma mandarin oranges that you are going to choose are of the goku-wase variety, which grow in gardens. The distance between the place of harvest and the store selling the oranges affects the amount of CO_2_ that is emitted. For example, the closer the proximity of the selling location is to the place of harvest, the lower the amount of CO_2_ emissions is and vice versa.

The fifth line provides space for you to indicate your decision. Please tick in the square that corresponds to the Satsuma mandarin orange of your choice. So, if you choose Satsuma mandarin orange A, please indicate the same in the corresponding square.

The last line provides the column for you to indicate the reason for your choice. The reasons for the choice consist of four factors: price, the CO_2_ emissions, the appearance of the Satsuma mandarin orange, and others. Please tick inside the square that corresponds to the reason why you have selected the particular Satsuma mandarin orange. For example, if you choose price as the reason, you should tick the square in the price column. Finally, close the box and wait for the experimenter to collect it.

The experimenter will collect all of the boxes in the room. This completes round one. The rules in round 2 are exactly the same as those in round 1. Initially, you receive 120 JPY, and then, you receive a box containing three types of Satsuma mandarin oranges and a record sheet. You purchase one of the three types of Satsuma mandarin oranges. After the completion of round 2, round 3 begins. This experiment is repeated a total of twelve times following the same rules. The completion of round 12 signals the end of the experiment.

**Earnings**

Earnings are calculated as the amount equal to the sum of the participation fee and total of the remaining amounts in twelve rounds. The participation fee is 500 JPY. Since this amount is a reward for your participation, it is not affected by your choices in each round.

Next, we explain the remaining amounts in the six rounds. At the beginning of each round, you receive 120 JPY to buy one Satsuma mandarin orange. The remaining amount in each round is equal to the difference between 120 JPY and the price of the Satsuma mandarin orange you choose. This amount constitutes your earnings in each round. Since this experiment consists of six rounds, you receive the sum of the remaining amount for six rounds. The formula for your earnings in the experiment is provided below.

Earnings = 500 JPY (show-up fee)

+ {(120 JPY – the price of the Satsuma mandarin orange that you buy in round 1)

+ (120 JPY – the price of the Satsuma mandarin orange that you buy in round 2)

+...+ (120 JPY – the price of the Satsuma mandarin orange that you buy in round 12)}

You need not be conscious of others because we never offer your earnings to others. This concludes the explanation of the experiment. Please understand the rules of the experiment and select the Satsuma mandarin orange that you wish to purchase.

Are there any questions before we begin?
