# Supplementary material for: Testing hypothetical bias in a choice experiment: An application to the value of the carbon footprint of mandarin oranges
Source: PLoS One. 2022 Jan 18;17(1):e0261369. doi: 10.1371/journal.pone.0261369 (PMC8765649; doi:10.1371/journal.pone.0261369)
Supplement: S2 File — (DOCX) [file pone.0261369.s003.docx]

**The cheap-talk script used in online**

Recent research shows that respondents in questionnaires tend to react differently to actual behavior.

For example, there is a difference between the amount that a respondent actually pays when buying a product in a store and the amount that he or she pays in the survey responses. There are various reasons why this difference can occur. One is that in a hypothetical situation, it will be difficult for respondents to imagine the actual texture and appearance of the goods. Also, it may be difficult for respondents to imagine that buying something will cause money to loose from their wallet. Also, it may be difficult for respondents to imagine that they loose money from their wallet to but something.
