# Supplementary material for: Testing hypothetical bias in a choice experiment: An application to the value of the carbon footprint of mandarin oranges
Source: PLoS One. 2022 Jan 18;17(1):e0261369. doi: 10.1371/journal.pone.0261369 (PMC8765649; doi:10.1371/journal.pone.0261369)
Supplement: Attachment — Submitted filename: Review PONE-D-21-18475.docx [file pone.0261369.s005.docx]

The authors investigate the issue of Hypothetical Bias in online Choice Experiments in a case study involving choices among oranges with different environmental impact. I think the paper is of interest both for the methodological aspect and the empirical case study and a valuable contribution to the literature. However, I think it needs to be revised before being suitable for publication, especially as it concerns the econometric analysis of choices. Below my comments.

- The transition between the review on carbon footprint studies and the outline of paper structure (lines 107-108) feels rather abrupt. I would add a sentence summarizing the findings of the literature and explaining what the authors expected to find in their study based on previous ones.
- I may have missed it, but I could not find the indication of the year in which the lab experiments took place. Given that the online surveys were carried out in 2013, I imagine several years could have passed between the surveys and the lab experiments. If that is the case, I think there should be a short discussion in the paper on whether this could have influenced the results.
- How were the priors for the D-efficient design retrieved?
- The issues of having a random cost coefficient in RPL models is poorly discussed in lines 298-304. You can refer to Mariel et al. (2021) for a recent discussion on this.

First of all, the cost coefficient "needs" to be negative for results to be consistent with the economic theory, as a positive coefficient would imply that utility increases when cost increases. Secondly, the actual issue with a randomly distributed cost coefficient is the possibility of retrieving positive tails of the distribution (with the above issue) and tails extremely close to zero, which make WTP values explode. At this point of the literature, I would not say that the conventional way to bypass such issues is a fixed cost coefficient, as the authors state. A fixed cost coefficient is criticizable because it implies the assumption that all respondents have the same marginal utility of income (very unrealistic). A much more common solution, nowadays, is to use a lognormal distribution with changed sign (to avoid positive tails), which still has the problem of possible points of the distribution very close to zero. This can be further avoided by adopting specifications in WTP-space (Train and Weeks 2005; Scarpa et al. 2008).

- Given the above and that the main purpose of the study is to compare WTP values across treatments, I would advise to estimate the models in WTP-space rather than in preference space, as models in WTP-space have been consistently found to yield more reliable WTP estimates (again see Mariel et al. (2021), chapter 6, for a recent discussion on this). I also note that nowadays is uncommon to use a normal distribution for cost (as done by authors) for the issues discussed in the previous point, but given that no positive tails were retrieved (based on Figure 4) it should not be an issue in this case. However, if you decide to change to a WTP-space specification, I would advise to use a log-normal distribution for the price/scale parameter.
- I find interesting that most of the covariates used to explain heterogeneity via interactions have significant effect only in some treatments, i.e. the effect is inconsistent across treatments. I think this deserves some discussion in the paper.
- Also about the covariates, I find surprising that low-income earners prefer cheaply priced oranges only in the hypothetical online setting, where no real money is involved. How would the authors explain this?
- The writing could use some improvements, there are several poorly constructed sentences throughout the text.

**References**

Mariel, P., Hoyos, D., Meyerhoff, J., Czajkowski, M., Dekker, T., Glenk, K., Jacobsen, J.B., Liebe, U., Olsen, S.B., Sagebiel, J., Thiene, M. 2021. Environmental Valuation with Discrete Choice Experiments. Guidance on Design, Implementation and Data Analysis. Springer, Cham.

Scarpa R, Thiene M, Train K (2008) Utility in willingness to pay space: a tool to address confounding random scale effects in destination choice to the Alps. Am J Agr Econ 90:994–1010.

Train K, Weeks M (2005) Discrete choice models in preference space and willingness-to-pay space. In: Scarpa R, Alberini A (eds) Springer. The Netherlands, Dordrecht, pp 1–16.
